# Cognitive Deficits in Parkinson's Disease Are Associated with Neuronal Dysfunction and Not White Matter Lesions

**DOI:** 10.1002/mdc3.13792

**Published:** 2023-05-29

**Authors:** Nils Schröter, Tobias Bormann, Michel Rijntjes, Ganna Blazhenets, Raissa Berti, Bastian E.A. Sajonz, Horst Urbach, Cornelius Weiller, Philipp T. Meyer, Alexander Rau, Lars Frings

**Affiliations:** ^1^ Department of Neurology and Clinical Neuroscience, Medical Center, Faculty of Medicine University of Freiburg Freiburg Germany; ^2^ Department of Nuclear Medicine, Medical Center, Faculty of Medicine University of Freiburg Freiburg Germany; ^3^ Department of Stereotactic and Functional Neurosurgery, Medical Center, Faculty of Medicine University of Freiburg Freiburg Germany; ^4^ Department of Neuroradiology, Medical Center, Faculty of Medicine University of Freiburg Freiburg Germany; ^5^ Department of Diagnostic and Interventional Radiology, Medical Center, Faculty of Medicine University of Freiburg Freiburg Germany; ^6^ Center for Geriatrics and Gerontology Freiburg, Medical Center, Faculty of Medicine University of Freiburg Freiburg Germany

**Keywords:** white matter lesion, cognition, neurodegeneration, Parkinson's disease, cognitive decline, white matter hyperintensities

## Abstract

**Background:**

Cognitive deficits considerably contribute to the patient's burden in Parkinson's disease (PD). While cognitive decline is linked to neuronal dysfunction, the additional role of white matter lesions (WML) is discussed controversially.

**Objective:**

To investigate the influence of WML, in comparison to neuronal dysfunction, on cognitive deficits in PD.

**Methods:**

We prospectively recruited patients with PD who underwent neuropsychological assessment using the Mattis Dementia Rating Scale 2 (DRS‐2) or Parkinson Neuropsychometric Dementia Assessment (PANDA) and both MRI and PET with [^18^F]fluorodeoxyglucose (FDG). WML‐load and PD cognition‐related covariance pattern (PDCP) as a measure of neuronal dysfunction were read out. Relationship between cognitive performance and rank‐transformed WML was analyzed with linear regression, controlling for the patients’ age. PDCP subject scores were investigated likewise and in a second step adjusting for age and WML load.

**Results:**

Inclusion criteria were met by 76 patients with a mean (± SD) age of 63.5 ± 9.0 years and disease duration of 10.7 ± 5.4 years. Neuropsychological testing revealed front executive and parietal deficits and a median DRS‐2 score of 137 (range 119–144)/144 and PANDA score of 22 (range 3–30)/30. No association between WML and cognition was observed, whereas PDCP subject scores showed a trend‐level negative correlation with the DRS‐2 (*P* = 0.060) as well as a negative correlation with PANDA (*P* = 0.049) which persisted also after additional correction for WML (*P* = 0.039).

**Conclusion:**

The present study indicates that microangiopathic WML do not have a relevant impact on neurocognitive performance in PD whereas neuronal dysfunction does.

Parkinson's disease (PD) is the most common neurodegenerative movement disorder and its relevance is expected to further increase due to demographic change.^1^ In addition to the disease‐defining motor deficits—rigor, tremor, bradykinesia and postural instability—non‐motor symptoms are receiving increased attention.^2^, ^3^ These comprise a wide range of symptoms, from olfactory disturbances, autonomic symptoms like constipation and pain to cognitive decline.

Cognitive deficits are frequently observed, with 46% of all PD patients developing dementia within 10 years after initial diagnosis, and about 90% of patients being affected during the entire disease course.^4^ This has far‐reaching socio‐economic consequences for patients and caregivers as well as for society, being a common factor for admission to nursing homes. Moreover, cognitive decline is associated with significantly reduced survival.^4^, ^5^, ^6^, ^7^

A variety of neuropathological studies identified cortical Lewy body‐related pathology as the leading pathological correlate of cognitive decline in PD, whereas Alzheimer's disease‐like co‐pathology was additionally observed in up to half of PD patients with dementia.^8^ Additionally, dopaminergic and cholinergic cortical denervation is linked to cognitive impairment in PD‐associated mild cognitive impairment (PD‐MCI) and PD‐associated dementia (PDD).^9^

In the early stages of cognitive decline, deficits in specific cognitive domains—especially executive function, working memory, and attention—can be observed in about one third of the patients, sometimes even preceding the onset of motor symptoms.^10^, ^11^ Apart from this frontostriatal, dysexecutive syndrome, a posterior cortical syndrome often develops that predicts dementia and comprises visuospatial and episodic memory deficits.^10^, ^11^, ^12^ These deficits are reflected by correlates in neuroimaging. In particular, the metabolic cognition‐related spatial covariance pattern in PD (PDCP) was elaborated using scaled subprofile model/principal component analysis (SSM‐PCA) on PET with [^18^F]fluorodeoxyglucose (FDG) in non‐demented patients with PD.^13^ PDCP is a covariance network characterized by metabolic reductions predominantly in medial frontal and parietal association areas and, to a lesser extent, increases in dentate nuclei and cerebellar vermis. Abnormally high PDCP expression (and hence frontal and parietal hypometabolism) was linked to cognitive deficits in PD.^13^, ^14^ The PDCP is suggested to reflect abnormalities in a variety of cognitive functions within the parkinsonian dementia syndrome and to indicate evolving cholinergic deficits. Increases in cerebellar and dentate metabolism were proposed to represent compensatory responses to the loss of dopaminergic input to the striatum.^13^

White matter lesions (WML) are commonly observed in the elderly population, with high lesion burden and strategic localizations leading to impaired cognitive abilities, especially executive functions, and potentially to vascular dementia.^15^, ^16^ In PD, WML are rather frequent, associated with supine hypertension and orthostatic hypotension, and correlate with an increased risk of developing PD‐MCI and PDD.^17^, ^18^ However, the role of WML as a potentially modifiable factor of cognitive decline in PD remains controversial, particularly since WML‐related deficits are quite similar to early cognitive deficits in PD. In an analysis of 108 patients with PD and 2 years of follow‐up considering WML and central cholinergic pathways, no association was found between WML and cognitive decline.^19^ In contrast, another study observed a significant association between WML and cognitive decline.^20^

We aimed to investigate the impact of WML on cognitive performance in comparison to neuronal dysfunction. Hence, we assessed WML load on cerebral MRI and brain glucose metabolism on FDG PET (using the PDCP score) and tested for associations with cognitive performance.

## Methods

### Participants

We report data from the bicentric FREE‐PD registry, which prospectively recruits patients with suspected parkinsonian disorders and/or dementias, who were admitted to the Department of Neurology, Medical Center–University of Freiburg between 3/2016 and 4/2022. Inclusion criteria for this study were the diagnosis of clinically established PD by two movement disorder specialists (MR; NS) according to current diagnostic criteria (3), a standardized neuropsychological assessment in ON‐state by Mattis Dementia Rating Scale 2 (DRS‐2)^21^ or Parkinson Neuropsychometric Dementia Assessment (PANDA)^22^ and both MRI and FDG PET scans with a maximum delay of 6 months. These examinations were typically carried out for differential diagnosis and to evaluate the best possible intensified treatment option for the patients (eg, before deep brain stimulation). DRS‐2 and PANDA were administered by a trained and experienced neuropsychologist (TB). These batteries examine the main cognitive domains: executive functions, learning, attention, spatial processing, and working memory. Motion artifacts and structural lesions (subdural hematoma, neoplasm or infarcts) on MRI data led to exclusion.

Scores on the DRS‐2 subtests were used to calculate a total score as well as subscores for different cognitive domains. In the case of PANDA subtests, raw scores were transformed into an ordinal scale, stratified by age.^22^ For both batteries, a total score was calculated with a maximum of 144 points for DRS‐2 and 30 points for PANDA.

### Image Acquisition and Preprocessing

**Figure 1:**
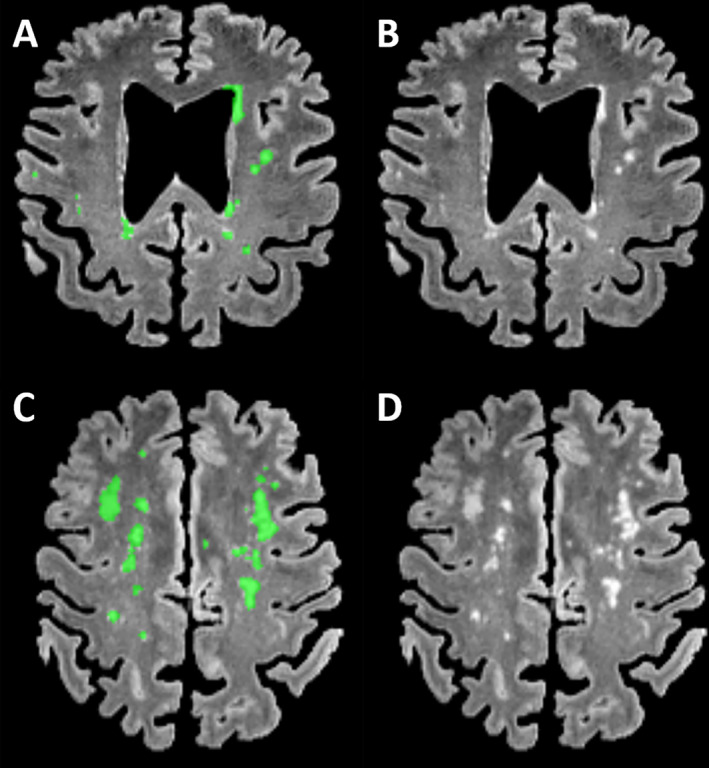
Axial FLAIR reformats in patients (A/B, 67 year‐old male; C/D, 75 year‐old female) with corresponding superimposed segmentations of white matter lesions (B, 4.8 ml total lesion load; D, 17.8 ml total lesion load).

MRI was performed with a 3 Tesla scanner (MAGNETOM Prisma, Siemens Healthcare, Erlangen, Germany) using a 64‐channel head and neck coil. T1‐weighted images were acquired with a three‐dimensional (3D) magnetization‐prepared 180° radio‐frequency pulses and rapid gradient‐echo (MP‐RAGE) sequence (repetition time: 2000 ms, echo time: 2.99 ms, flip angle: 8°, TI = 1010 ms, GRAPPA factor = 2, 0.8 mm isotropic voxels, 192 contiguous sagittal slices, bandwidth: 240 Hz/pixel). The 3D T2‐weighted FLAIR (fluid attenuated inversion recovery) sequence was acquired with the following parameters: repetition time: 5000 ms, echo time: 388 ms, flip angle: variable, TI = 1800 ms, GRAPPA factor = 2, 1.0 mm isotropic voxels, 192 contiguous sagittal slices. White matter T2w hyperintensities were manually segmented on 3D reformatted T2w‐FLAIR images as depicted in Figure 1 according to the literature by an experienced radiologist (AR).^23^ Total WML load of each individual patient was subsequently read out as volume (ml). WML were normalized on total intracranial volume (TIV) derived by the CAT12 (http://www.neuro.uni-jena.de/cat/); tissue compartment segmentation in Statistical Parametric Mapping (SPM12, https://www.fil.ion.ucl.ac.uk/spm/) and rank‐transformed to address the skewed distribution.

FDG PET scans were acquired on either Philips Gemini or Vereos PET/CT systems (Philips, The Netherlands). Patients fasted for at least 6 h before intravenous injection of 255 ± 76 (Philips Gemini) or 211 ± 18 MBq FDG (Philips Vereos) under resting conditions at ambient noise and with eyes open. A static 10‐min image was acquired 50 min post injection. Fully corrected FDG PET datasets were reconstructed as previously described^24^, ^25^ and spatially normalized to an in‐house FDG PET template in Montreal Neurological Institute space using SPM12. The spatially normalized FDG PET dataset was scaled to the average FDG uptake of brain parenchyma (mask from SPM tissue probability map with probability for both gray and white matter of at least 50%). To harmonize the spatial resolution in the reconstructed images, Gaussian filters of various kernels were applied to each dataset to achieve an isotropic final resolution of 9 mm. As a metabolic marker of cognitive function in PD patients, we assessed the expression of the metabolic spatial covariance pattern (PDCP) associated with cognitive function in PD developed by Huang and colleagues [LIT 13 Huang et al]. PDCP is a well‐established marker of regional metabolism that has previously shown a high predictive value in PD with cognitive decline. To calculate individual PDCP scores for this cohort, we employed topographic profile rating algorithm using the ScAnVP/SSM/PCA toolbox (The Feinstein Institute for Medical Research) as previously described.^26^ For reference, PDCP scores were also computed in FDG PET data of a group of 48 healthy elderly controls acquired on Philips Vereos PET/CT (n = 13) and Philips Gemini PET/CT (n = 35). The PDCP scores of PD patients were z‐transformed using the PDCP scores of the control group.

### Statistical Analysis

Statistical analysis was performed with R software (version 4.0.3, http://www.R-project.org). The significance threshold was set to *P* = 0.05.

1. WML volume was tested for associations with the test scores of PANDA and DRS‐2 separately by linear regressions controlling for age.
2. The Z‐transformed PDCP subject score was tested for associations with the test scores of PANDA and DRS‐2 separately, controlling for age, *with* and *without* WML load as covariate.
3. In exploratory analyses, WML load and Z‐transformed PDCP subject scores were tested for associations with DRS‐2 subscales and PANDA subtest scores by linear regressions controlling for age.

All analyses were performed both in the total cohort as well as stratified by severity of cognitive deficits into the groups with MCI (score below 140), dementia (score below 133), and cognitively normal with a score of at least 140.

We conducted a post‐hoc power analysis for detecting an association between cognition and WML using G*Power 3.1.^27^

## Results

### Participants

In total, 76 patients were eligible for inclusion in this study. They underwent DRS‐2 (n = 75/76) or PANDA (n = 72/76) and both MRI and FDG PET as part of clinical routine between March 2016 and April 2022. Mean (± standard deviation; SD) age was 63.5 ± 9.0 years, 27 (36%) patients were female and the mean disease duration was 10.7 ± 5.4 years. For further patient characteristics see Table 1. The median delay was 1.5 days (interquartile range (IQR): 4.25) between both scans, 3 days (IQR: 5) between neuropsychological testing and MRI, and 4 days (IQR: 5) between neuropsychological testing and FDG PET. In 39 (51%) patients, a [^123^I]FP‐CIT SPECT was also available, which confirmed pathologically reduced availability of striatal dopamine transporter (DAT) in each case.

**TABLE 1:** Demographic characteristics of patient group

	Overall	DEM	MCI	NC
n	76	17	36	23
Age (years) (mean (SD))	63.46 (8.97)	67.07 (6.52)	63.64 (9.97)	60.50 (8.12)
Gender (Female) (%)	27 (35.5)	4 (23.5)	14 (38.9)	9 (39.1)
Hoehn & Yahr Stage (median [IQR])	3.00 [2.00, 3.00]	3.00 [3.00, 3.00]	3.00 [2.00, 3.00]	2.50 [2.00, 3.00]
UPDRS‐III‐Score (mean (SD))	47.41 (18.15)	53.81 (17.86)	44.61 (16.02)	47.35 (20.96)
Levodopa equivalent dose (mean (SD))	861.05 (371.71)	877.13 (458.01)	854.00 (352.10)	860.20 (347.77)
Symptom Duration (years) (mean (SD))	10.66 (5.44)	12.78 (6.42)	9.87 (5.07)	10.34 (5.03)
Duration of clinical Follow‐up after Examination (years) (mean (SD))	1.46 (1.40)	1.37 (1.93)	1.24 (1.21)	1.88 (1.16)
White‐Matter‐Lesion Load (ml) (median [IQR])	1.22 [0.24, 2.63]	1.93 [0.33, 2.65]	1.67 [0.32, 2.74]	0.40 [0.03, 2.09]
PDCP scores (z) (mean (SD))	1.39 (1.10)	1.87 (1.33)	1.32 (1.02)	1.13 (0.98)
Years of Education (mean (SD))	13.11 (3.29)	11.93 (3.56)	13.40 (3.33)	13.45 (2.99)
Dementia Rating Scale 2 (DRS2)—Total Score (median [IQR])	137.00 [133.00, 141.00]	130.00 [129.00, 131.00]	136.00 [135.00, 138.50]	142.00 [141.00, 143.00]
Attention Subscale (median [IQR])	36.00 [36.00, 37.00]	36.00 [35.00, 36.00]	36.00 [36.00, 37.00]	37.00 [36.00, 37.00]
Initiation/Perseveration (median [IQR])	36.00 [32.00, 37.00]	31.00 [29.00, 32.00]	35.00 [33.00, 37.00]	37.00 [37.00, 37.00]
Construction Subscale (median [IQR])	6.00 [6.00, 6.00]	6.00 [6.00, 6.00]	6.00 [6.00, 6.00]	6.00 [6.00, 6.00]
Conceptualization Subscale (median [IQR])	37.00 [36.00, 38.00]	36.00 [35.00, 37.00]	37.00 [35.50, 38.00]	39.00 [37.00, 39.00]
Memory Subscale (median [IQR])	23.00 [22.00, 24.00]	22.00 [21.00, 22.50]	23.00 [22.00, 24.00]	24.50 [23.25, 25.00]
PANDA—Total Score (median [IQR])	22.00 [19.00, 26.00]	20.00 [13.00, 22.50]	22.00 [19.00, 25.75]	25.00 [22.00, 28.00]
Paired‐Associate‐Learning (mean (SD))	7.34 (2.72)	6.43 (3.92)	7.64 (2.16)	7.48 (2.58)
Verbal Fluency (mean (SD))	13.40 (3.74)	9.79 (2.12)	13.24 (3.27)	16.05 (3.22)
Spatial Processing (median [IQR])	2.00 [1.75, 2.25]	1.50 [1.00, 2.00]	2.00 [2.00, 2.00]	2.00 [2.00, 3.00]
Working Memory (median [IQR])	5.00 [4.00, 6.00]	5.00 [4.00, 5.00]	5.00 [4.00, 6.00]	5.00 [5.00, 6.00]
Delayed Recall (median [IQR])	3.00 [2.00, 4.00]	2.50 [1.25, 4.00]	3.00 [2.00, 3.00]	3.00 [2.00, 4.00]

Note: Data are presented as mean value ± SD or median and IQR. Continuous data are presented as median and range or mean and standard deviation as appropriate.

Abbreviation: DEM, patients classified as suffering from dementia according to DRS‐2 < 133; DRS‐2, Mattis Dementia Rating Scale 2; IQR, interquartile range; MCI, patients classified as suffering from mild cognitive impairment according to DRS‐2 < 140, >132; NC, patients classified as cognitively normal according to DRS‐2 > 139; PANDA, Parkinson Neuropsychometric Dementia Assessment; PDCP, Parkinson's disease cognition pattern; SD, standard deviation; UPDRS‐III, Unified Parkinson's Disease Rating Scale Part 3.

### Neuropsychology

According to DRS‐2 thresholds by Matteau et al,^21^ 35/75 patients (47%) had MCI (score below 140), 17/75 (23%) had dementia (score below 133), and 23/75 (31%) had a normal score of at least 140. One patient who was not examined with the DRS‐2 had a PANDA total score of 15, which according to Kalbe et al^22^ indicates “subtle cognitive disturbance”.

**Figure 2:**
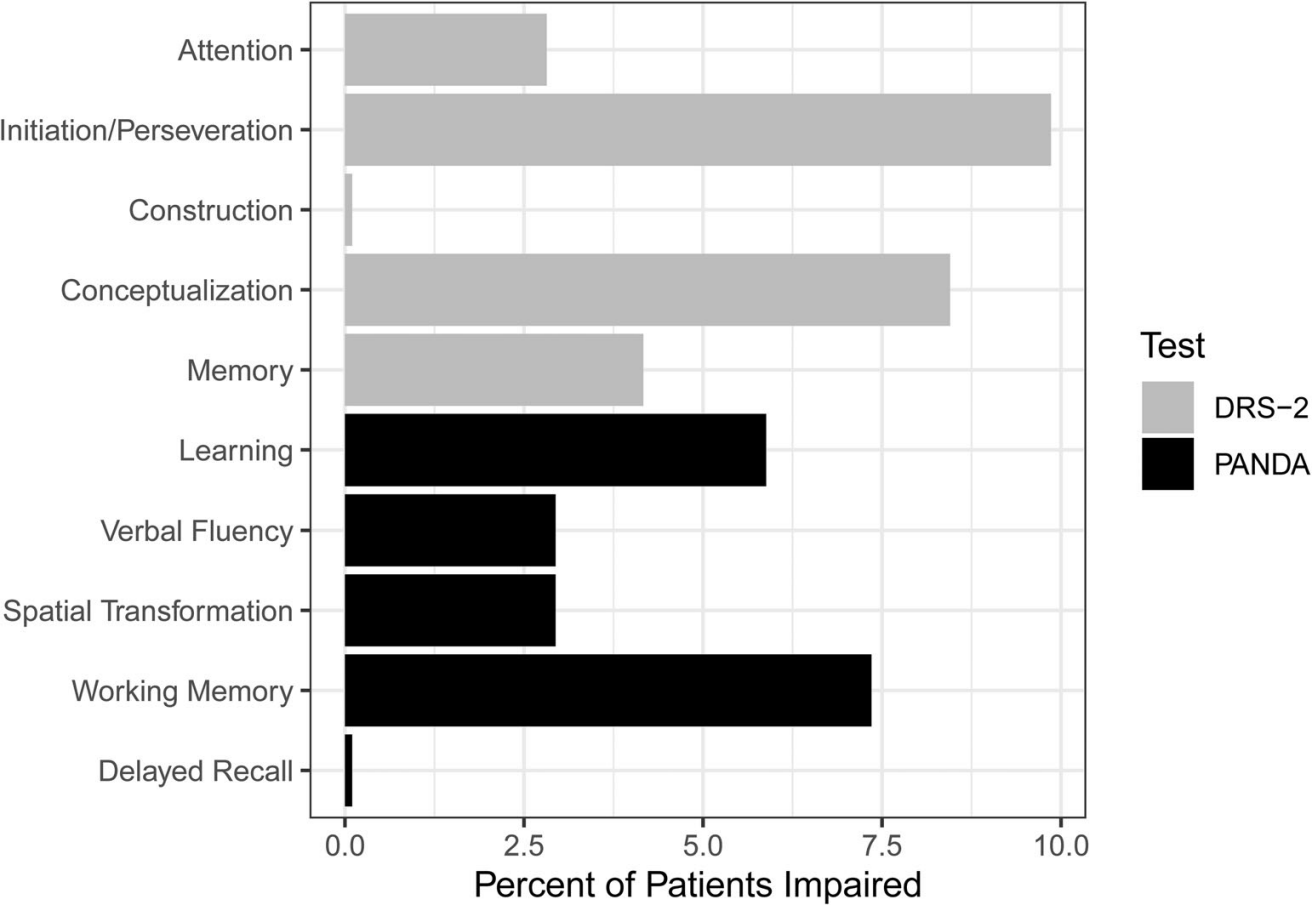
Percent of patients impaired in Mattis Dementia Rating Scale 2 (DRS‐2) subscales and Parkinson Neuropsychometric Dementia Assessment (PANDA) subtests (based on normative data from Bezdicek et al^28^ and Gasser et al^29^).

DRS‐2 subscales with the highest proportions of impaired patients (scores below the 2nd percentile according to education‐adjusted normative data by Bezdicek et al^28^) were the initiation/perseveration (7/71 patients, 10%) and conceptualization (6/71, 8%) subscales. For PANDA, impairment (education‐adjusted scores more than 2 SD below the mean of healthy individuals^29^) was predominantly observed in working memory (5/68 patients, 7% of patients impaired) and learning subtests (4/68 patients, 6%; for details, see Fig. 2).

### Neuroimaging

**Figure 3:**
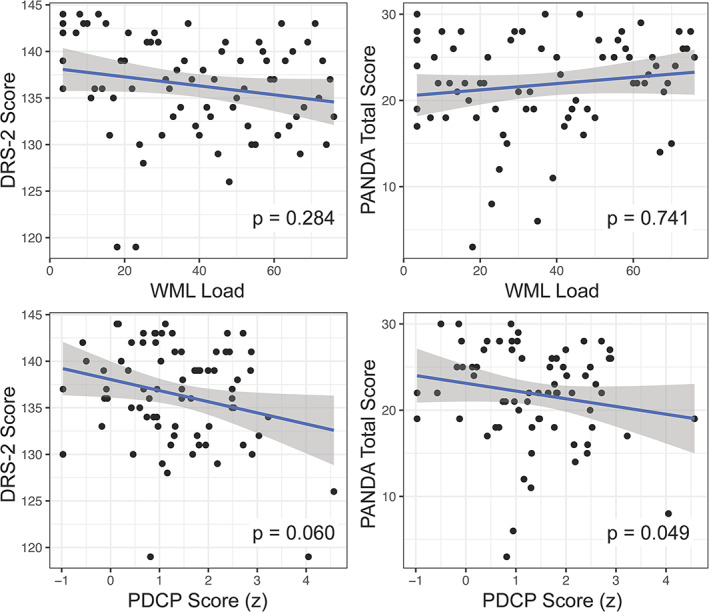
Association between cognitive performance (DRS‐2 and PANDA) and neuroimaging. WML load values are normalized to intracranial volumes and rank‐transformed. DRS‐2, Mattis Dementia Rating Scale 2, Parkinson Neuropsychometric Dementia Assessment; PDCP, PD‐cognition‐related metabolic pattern.

Patients had a median total WML load of 1.2 ml with a range from 0 to 25 ml. No additional vascular pathologies (eg, cortical or lacunar infarction) were observed. Z‐transformed PDCP scores of the patients ranged from −1.0 to 4.6 and were at least slightly increased (1 SD above the mean of the control group) in 46/76 (61%) and severely increased (2 SD above the mean of the control group) in 22/76 (29%) (see Fig. 3).

### Associations between Cognitive and Imaging Parameters

1. No significant associations (controlled for age) between WML load and total scores of DRS‐2 (*t* = −1.08; *P* = 0.284) or PANDA (*t* = 0.331; *P* = 0.741) were observed. For distribution and association with PANDA or DRS‐2 see Fig. 3 (top row). Furthermore, no significant associations were found when this analysis was repeated replacing rank‐transformed and TIV‐normalized WML load by raw WML load volume, TIV‐normalized WML load, or log‐transformed TIV‐normalized WML load (see Fig. S1).
2. Z‐transformed PDCP subject scores showed a trend‐level negative association (controlled for age) with the DRS‐2 (*t* = −1.91, *P* = 0.060) as well as a significant negative association with PANDA (*t* = −2.01, *P* = 0.049; Fig. 3, bottom row) that also persisted after additional correction for WML load (DRS‐2: *t* = −1.73, *P* = 0.087; PANDA: *t* = −2.11, *P* = 0.039).
3. In exploratory analyses, no significant association (controlled for age) was observed between WML load and cognitive domains of DRS‐2 and PANDA, only a trend‐level positive association (*t* = 1.68, *P* = 0.098) was found between WML load and PANDA delayed recall. Z‐transformed PDCP subject scores were associated with the PANDA spatial transformation scores (*t* = −2.07, *P* = 0.043) and the DRS‐2 attention subscale (*t* = −1.97, *P* = 0.053), controlled for age. Associations between PDCP subject scores on one side and PANDA spatial transformation scores or the DRS‐2 attention subscale on the other were essentially unaltered after additionally controlling for WML load (PANDA spatial transformation: *t* = −2.06, *P* = 0.044; DRS‐2 attention: −1.93, *P* = 0.059).

### Power Analysis for Detecting Associations between WML Load and Cognition

From the literature, a wide span of effect sizes can be derived: In the meta‐analysis by Liu et al,^30^ significant WML load differences between age‐matched PDD and non‐demented PD were reported with effect sizes (Cohen's *d*) of 0.52 to 1.23. Notably, studies on PD‐MCI versus PD with normal cognition (age‐matched groups) did not reveal a significant difference (Cohen's *d* of 0.15 and 0.04). Dadar et al reported correlations (Pearson's r) between WML load and cognitive measures at baseline of about −0.2.^20^ For our study, power analyses (at alpha = 0.05 and *N* = 76) for small to medium (*r* = 0.2), medium (*r* = 0.3), and large effects (*r* = 0.5) revealed 55%, 86%, and 99.96% power.

## Discussion

The present study on a cohort of 76 well‐characterized patients with PD provides evidence that cognitive deficits in PD are not associated with WML, but with neuronal dysfunction.

The PDCP comprises metabolic decreases in the frontal and parietal cortex, and cerebellar increases.^13^ Decreases in PDCP overlap with the typical hypometabolic regions in cognitively impaired patients with PD.^12^, ^31^ Hence, we interpret elevated PDCP scores to mainly reflect frontal and temporo‐parietal hypometabolism and to indicate neuronal dysfunction.

So far, the effect of WML on cognitive performance in PD is controversial. In a study by Rane et al,^32^ the burden of WML in PD was associated with poor performance on a test of executive functioning (category fluency). Patients in that study, however, were substantially older compared to our study. Thus, a potentially higher overall WML load, among other differences from our own study, could explain this discrepant finding. Other studies showed a prognostic value of WML load in PD for the later development of dementia^33^ or steeper cognitive decline.^20^ Results from the two studies indicate a detrimental effect of WML load on future cognitive performance in PD. However, baseline cognitive performance did not correlate with baseline WML load, which is consistent with our results. Also in agreement with our study, Hanning et al^19^ found no association between WML load and worse cognitive performance in PD. Liu et al^30^ presented a meta‐analysis of nine studies in non‐demented patients with PD. They concluded that WML burden did not differ between PD patients with MCI and those with normal cognition when controlling for age, which is also in line with our findings.

The average cognitive impairment of patients in our study was mild, and the majority of patients had MCI, although a relevant subset suffered from dementia. Of note, mild impairment nevertheless might have an impact on everyday life and displayed the typical profile of (early in the disease course) “anterior,” frontostriatal deficits (executive function, attention) and (often developing later in the disease course) “posterior,” temporo‐parietal deficits (visuospatial functions, memory).^11^, ^12^ In an exploratory analysis, we observed an association between the PDCP subject score and performance in the visuospatial domain. This is consistent with the initial publication of Huang et al.^13^ However, this association was only significant at a liberal threshold (not corrected for multiple tests). In contrast to Huang et al,^13^ we found no associations between PDCP subject scores and executive function or memory performance.

WML load in our patients was rather mild, and an association between WML load and cognitive impairment might be more easily detected in cohorts with a higher WML load. However, the WML load of the present study (median: 1.2 ml, mean: 2.8 ml) is within the range of average WML volumes reported in earlier studies which assessed the potential influence of WML on cognition in PD (mean volumes in PD with normal cognition from 0.4 to 4.2 ml, in PD‐MCI from 0.4 to 12.3 ml).^30^

DRS‐2 detected a greater proportion of impaired patients than PANDA. This might suggest DRS‐2 to be more sensitive for screening cognitive impairment in patients with PD without subjective complains. However, the proportion of patients in whom cognitive impairment is detected, obviously depends on the chosen cut‐off and the cohort from which normative data were obtained. Due to a lack of cut‐offs for DRS‐2 and PANDA established in the same normative data cohort, we used cut‐offs from different cohorts, which hampers the comparison of sensitivity.

The cross‐sectional design can solely provide information on an association between WML and the magnitude of cognitive deficits, but not the rate of progression. Even though earlier studies worked with similar sample sizes, one cannot rule out the possibility that a statistically significant correlation between WML load and cognition might be discovered with a higher number of cases.^31^ However, our post hoc power analysis revealed that the number of cases assessed in this study was sufficient to detect medium and large effects. The absence of non‐motor symptom questionnaires is another limitation, preventing the assessment of the effect of WML on non‐motor symptoms. On the contrary, the present study has several strengths: First, the relevance of WML was investigated in a relatively large, well‐characterized cohort with considerable cognitive dysfunction. Second, the findings are consistent across two test batteries validated in the assessment of neuropsychological deficits in PD. Third, the impact of neuronal dysfunction on cognitive deficits was verified by assessing PDCP expression as a validated approach.^13^

The present study indicates that microangiopathic white matter lesions do not have a relevant impact on neurocognitive performance in Parkinson's disease, whereas neuronal dysfunction does.

## Author Roles

(1) Research Project: A. Conception, B. Organization, C. Execution; (2) Statistical Analysis: A. Design, B. Execution, C. Review and Critique; (3) Manuscript Preparation: A. Writing of the First Draft, B. Review and Critique.

N.S.: 1A, 1B, 1C, 2C, 3A.

T.B.: 1B, 1C, 2C, 3B.

M.R.: 1B, 3B.

G.B.: 1B, 1C, 3B.

R.B.: 1B, 1C, 3B.

B.S.: 1B, 3B.

H.U.: 1B, 3B.

C.W.: 1B, 3B.

P.M.: 1A, 1B, 2A, 2C, 3B.

A.R.: 1A, 1B, 1C, 2C, 3A.

L.F.: 1A, 1C, 2A, 2B, 2C, 3B.

## Disclosures

**Ethical Compliance Statement:** The register study FREE‐PD was approved by the local ethics committee of the Freiburg Medical Center (EK 100/19). Written, informed consent for this study was obtained and documented from all participants in accordance with the Declaration of Helsinki and its later amendments. We confirm that we have read the Journal's position on issues involved in ethical publication and affirm that this work is consistent with those guidelines.

**Funding Sources and Conflicts of Interest:** No specific funding was received for this work and the authors declare that there are no conflicts of interest relevant to this work.

**Financial Disclosures for the Previous 12 Months:** NS received grants from Berta‐Ottenstein‐Programme for Clinician Scientists, Faculty of Medicine, University of Freiburg, and honoraria from Abbvie (presentation). PTM received honoraria from GE (presentation, consultancy) and Philips (presentation). AR received grants from Berta‐Ottenstein‐Programme for Clinician Scientists, Faculty of Medicine, University of Freiburg. BEAS received a research grant from CereGate, Munich, Germany unrelated to this publication. All other authors have nothing to report. All declared interests are outside of the submitted work.

## Supporting information


**Figure S1.** WML load values as raw values; normalized to intracranial volume; normalized to intracranial volume and log‐transformed; normalized to intracranial volume and rank‐transformed. DRS‐2, Mattis Dementia Rating Scale 2, Parkinson Neuropsychometric Dementia Assessment.

## Data Availability

The data supporting the findings of this study are available upon reasonable request and approval of the local ethics committee.
