# Supplementary figures and images for: Cognitive Deficits in Parkinson's Disease Are Associated with Neuronal Dysfunction and Not White Matter Lesions
Source: Mov Disord Clin Pract. 2023 May 29;10(7):1066–73. doi: 10.1002/mdc3.13792 (PMC10354622; doi:10.1002/mdc3.13792)

**SUPPLEMENTAL RESULTS**


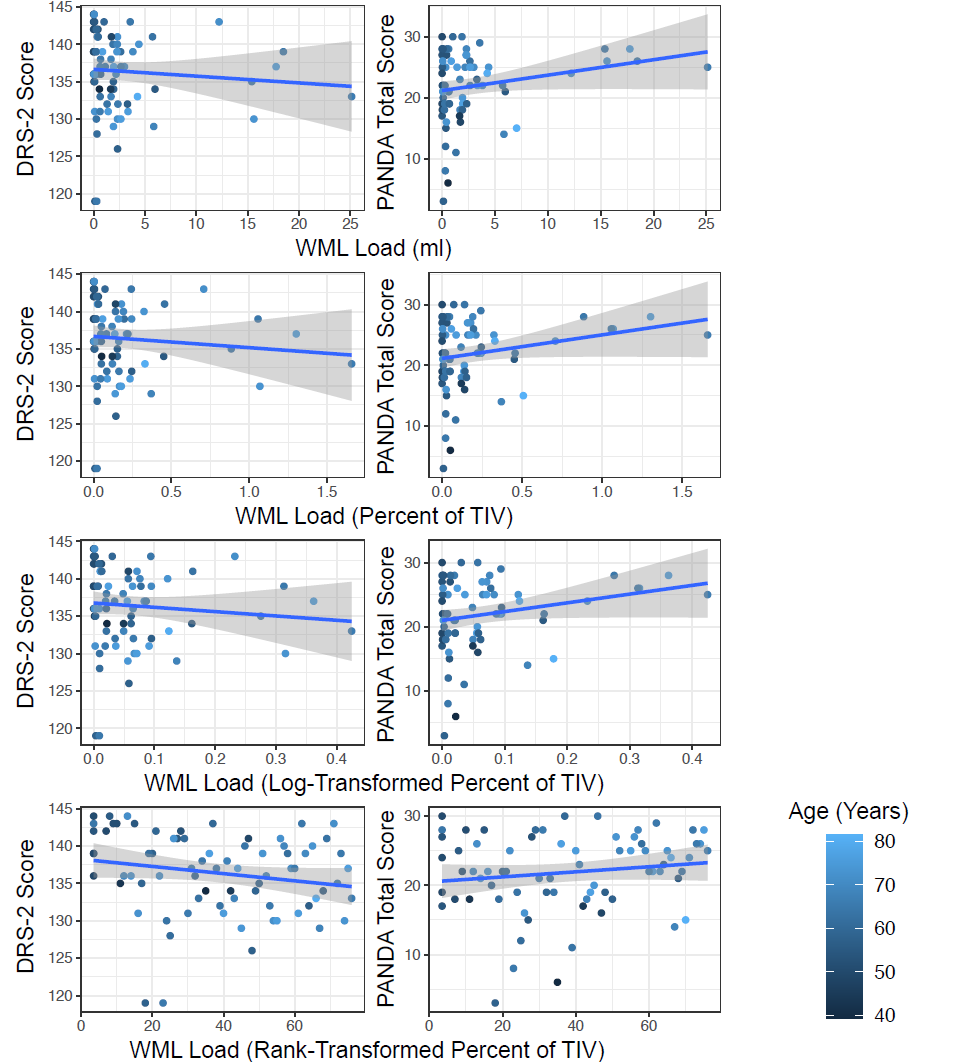


**Supplemental Figure 1**

Supplement: Supplementary file 1 — Figure S1. WML load values as raw values; normalized to intracranial volume; normalized to intracranial volume and log‐transformed; normalized to intracranial volume and rank‐transformed. DRS‐2, Mattis Dementia Rating Scale 2, Parkinson Neuropsychometric Dementia Assessment. [file MDC3-10-1066-s001.docx]
